# Economic impact of digital dermatitis, foot rot, and bovine respiratory disease in feedlot cattle

**DOI:** 10.1093/tas/txab076

**Published:** 2021-05-19

**Authors:** Julian Alberto Cortes, Steve Hendrick, Eugene Janzen, Ed A Pajor, Karin Orsel

**Affiliations:** 1 Faculty of Veterinary Medicine, Department of Production Animal Health, University of Calgary, Calgary, Canada; 2 Coaldale Veterinary Clinic, Coaldale, Canada

**Keywords:** beef cattle, BRD, economic impact, oot rot, infectious disease, lameness

## Abstract

Digital dermatitis (**DD**) has emerged in North American feedlots, although production and economic impacts are not fully understood. Objectives of this study were to: (1) estimate the economic impact of a single case of DD, foot rot (**FR**), and bovine respiratory disease (**BRD**) in feedlot cattle and (2) determine its impact on average daily gain (**ADG**). Feedlot cattle health and production records were available from two feedlots for a 3-yr interval. The dataset consisted of 77,115 animal records, with 19.3% (14,900) diagnosed with a disease. Diseased animals were categorized into five groups: DD, FR, BRD, other diseases (OT), and two or more diseases (TM), with a treatment cumulative incidence of 6.0%, 59.1%, 10.7%, 12.7%, and 11.5%, respectively. FR was the disease with the highest cumulative incidence in both heifers and steers (58.8% and 59.6%, respectively). Of all fall-placed cattle diagnosed with any disease, 48.1% of the cases were FR. DD affected the partial budget in five out of the eight groups of cattle, with the highest impact of DD seen in grass yearling heifers and grass yearling steers: $-98 and $-96 CAD, respectively, relative to their healthier counterparts. Healthy cattle had a significantly higher ADG when compared with DD cattle in five of the eight categories, ranging from 0.11 kg/d in winter-placed heifers to 0.17 kg/d in fall-placed steers. In the economic analysis, it was concluded that on an individual animal basis, BRD was the most impactful of all analyzed diseases, whereas DD was second, marking the importance of controlling and mitigating this foot condition. Identifying differential effects of diseases on a partial budget analysis and ADG of the types of cattle stratified by sex enables feedlot producers to focus control and mitigation strategies on specific groups.

## INTRODUCTION

Lameness, a clinical manifestation of an abnormal condition that affects locomotion (Van Nuffel et al., 2015), is the second most treated condition in feedlot cattle after bovine respiratory diseases (**BRDs**) (Davis-Unger et al., 2018), with adverse effects on animal health, production, and welfare. Foot lesions are responsible for 70–90% of lameness cases in both dairy (Solano et al., 2016) and beef cattle (Griffin et al., 1993; Schwartzkopf-Genswein et al., 2016), with infectious lesions such as digital dermatitis (**DD**), foot rot (**FR**), and interdigital dermatitis having the highest prevalence (Brown et al., 2000; Teixeira et al., 2010; Refaai et al., 2013).

DD is a multifactorial polybacterial contagious foot disease, with microorganisms that belong to the species *Treponema* consistently isolated from DD lesions (Krull et al., 2014; Zinicola et al., 2015). It was first reported in Italy in 1974 (Cheli, 1974) and subsequently in several countries around the world (Orsel et al., 2018), affecting both dairy (Solano et al., 2016) and beef cattle (Sullivan et al., 2013). In the former, it is usually present between heel bulbs of rear feet, where it can develop skin ulcers that cause discomfort or pain (Döpfer et al., 1997), although for beef cattle its manifestation is not as well characterized.

FR, also called interdigital necrobacillosis, causes subcutaneous swelling that results in sudden lameness, where the main bacterium associated with this disease is *Fusobacterium necrophorum* (John and Whittier, 2009). Excess of moisture and unhygienic environments have been considered to be predisposing factors in dairy cattle for most infectious foot lesions (Hultgren and Bergsten, 2001; Solano et al., 2017), whereas for beef cattle this has not been fully elucidated.

Lameness accounts for 16% of all morbidities in feedlot cattle and up to 70% of revenue losses related to premature slaughter (salvaged) cattle, mainly due to chronic injury, treatment, decreased average daily gain (**ADG**), and increased days on feed (**DOF**) (Terrell et al., 2017). Estimated costs have been reported for other feet and leg conditions (Davis-Unger et al., 2018), albeit not for DD. Although DD is regarded as causing clinical lameness in heavy cattle that are nearly finished and ready for market, it is not clear how impactful it is (Plummer and Krull, 2017).

However, Kulow et al. (2017) reported significantly lower carcass weight in beef cattle with active M2 lesions relative to those without M2 lesions.

No studies to date have performed a partial budget analysis (= benefit–cost) of healthy cattle (**HE**; not diagnosed as sick) to diseased cattle with a single DD incidence. To address this gap in knowledge and build on previous research, the objective was to estimate the economic and production impact of a single DD incidence, specific to various cattle types and both sexes. To provide a frame of reference, cattle with a single incidence of FR, BRD, or other and multiple diseases were also included.

## MATERIALS AND METHODS

This study used data from two finishing feedlots in southern Alberta with outdoor housing in pens, dirt floors, and wind protection as is common in Western Canada. The dataset was not purposively collected, and it was provided by Coaldale Veterinary Clinic (**CVC**), Lethbridge, Alberta, Canada with feedlot workers recording data in a computer software program (Fusion, SSG Fusion). Diagnosis and treatment of diseases were performed according to the protocols provided by CVC, in which a DD case was defined as a skin lesion between the heel bulbs that could be either active or chronic; a FR case as a symmetrical swelling of the foot with necrotic tissue between the toes and a foul odor; and a BRD case was defined as a depressed animal with a difficulty to breathe, coughing, nasal discharge, and fever once it was pulled.

Cattle in these feedlots were handled according to the Code of Practice for the Care and Handling of Beef Cattle (AAFC, 2018).

Data were available for a 3-yr interval (2016–2018) in which 34 different diseases were diagnosed. Stratification was done for cattle who had a single disease incidence of DD, FR, or BRD. Besides, two new categories were created: one for cattle that had a single incidence of any other disease (OT) and another for cattle that were pulled for two or more diseases (TM), including any disease category mentioned previously.

Each individual in the dataset had feedlot identification, type of cattle [fall-placed calves (**FPC**) placed between September 22 and December 20; winter-placed calves (**WPC**) placed between December 21 and March 19; grass-fed yearlings (**GY**, cattle placed on grass for the summer before going to a finishing feedlot); and background yearlings (**BY**, cattle placed in a feedlot with a moderate-energy diet before going to a finishing feedlot)], cattle identification, arrival date, arrival weight (**AW**), DOF, date of slaughter, hot carcass weight (**HCW**), sex (steer or heifer), marbling, yield, premium/discount (relative to meat quality), processing cost, and feed cost. Cattle that were treated had the following additional information: diagnosis, date of diagnosis and treatment, and treatment costs. DOF at diagnosis was calculated using date of diagnosis minus date at arrival.

### Data Cleaning

**Figure 1.**
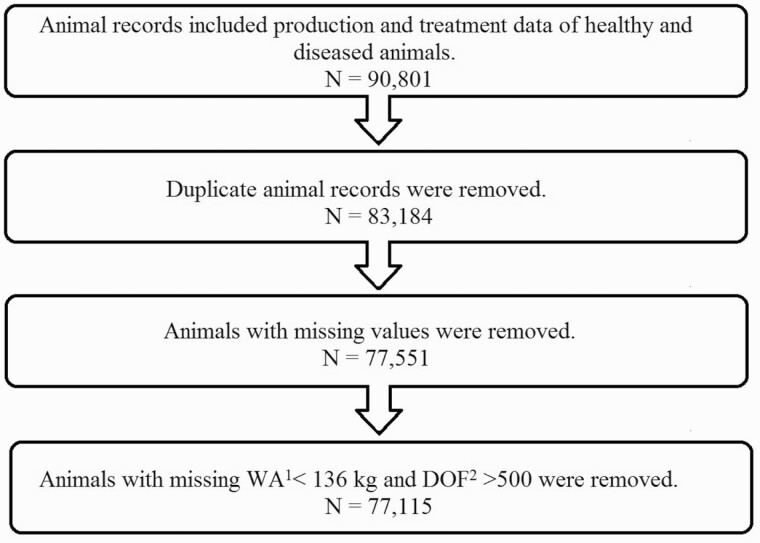
Diagram showing data filtering and cleaning of animal records. ^1^WA, weight at arrival; ^2^DOF, days on feed.

Identical copies of records were defined as duplicates and removed from the database. For missing values, treated cattle without production parameters or negative values were omitted (*n* = 5,033); similarly, for missing information on AW (*n* = 301), sex (*n* = 200), or type of cattle (*n* = 99). In addition, cattle with a weight at arrival <136 kg and DOF >500 (*n* = 436) were not included in the analysis, given their unlikely occurrence in the western Canadian feedlot setting, deeming them as a mistake in data entry as was suggested by Davis-Unger et al. (2018). This cleaning process resulted in exclusion of 13,686 animal records, resulting in *n* = 77,115 animal records for this study (Figure 1). One of the feedlots contributed 73% of the resulting number of available records.

Therefore, the variable weight at slaughter (**WS**) was calculated by dividing HCW by 0.596 (Bartoň et al., 2006). The weight at arrival (**WA**) was then discounted from WS and the result divided by the DOF to create the continuous variable ADG (kg/d):


$$\begin{array}{l} ADG=\frac{WS-WA}{DOF} \end{array}$$

### Economic Parameters

All economic results are reported in Canadian dollars ($CAD). For the partial budget analysis, only those variable benefits and costs as affected by the disease status were considered. We used the following formula [Eq. (1)]:


$$\begin{array}{l} partial\;budget=benefit-cost. \end{array}$$ (1)

The benefit per animal was calculated using the following formula [Eq. (2)]:


$$\begin{array}{l} Benefit=HCW*[BPM+(MQ)], \end{array}$$ (2)

where BPM stands for base price of meat and MQ for meat quality, categorized as premium or discount. As base price varies according to market volatility, the average was calculated for the 3-yr interval (2016–2018) and used as a constant for all cattle in the database (= $5.65CAD/HCWkg). The premium or discount value was calculated individually, based on meat quality of each animal, determined on slaughter information on yield and grade, thereby affecting the total revenue (Bureš and Bartoň, 2018).

To calculate cost, the following formula was used [Eq. (3)], with cost of calf at arrival not included due to a lack of data availability:


$$\begin{array}{l} cost=treatment+processing+feeding. \end{array}$$ (3)

All costs were obtained from the CVC dataset, in which they are reported on an individual animal basis. Treatment costs differed based on the protocol for the disease treated; processing cost varied according to the animal type, its weight, and the number of times it was processed through the chute; and finally feeding cost. As the economic analysis was done for a 3-yr interval, the cost of the feeding ingredients changed during that interval, thereby influencing the total cost of the ratio. Besides, the amount fed and DOF also influenced the total feeding cost. Therefore, costs included should reflect all events from arrival at the feedlot to departure to the abattoir and therefore indirectly reflect the risk type through inclusion of AW and DOF.

### Statistical Analyses

Data were analyzed using STATA 14.1 software (StataCorp LP, College Station, TX). Descriptive statistics were calculated for HE and each category of diseased cattle. Average, standard deviations, and confidence intervals were generated for variables that were normally distributed. Cattle were stratified by type (FPC, WPC, GY, BY), sex (heifer, steer), and disease status (HE, DD, FR, BRD, OT, or TM). As observations were nonpaired and data had a parametric distribution, an analysis of variance was used to compare means within stratified groups, followed by Scheffe’s post hoc test [i.e., HE fall-placed heifers (**FPHs**) vs. DD FPHs]. Correction for pen origin was not possible due to lack of information regarding resorting of cattle during the feeding cycle. Also, by stratifying for sex, data were adjusted for feedlot as well, as the two feedlots analyzed had either steers or heifers, but not both. Values were considered significantly different if *P* < 0.05.

## RESULTS

### Cumulative Incidence of Foot Lesions and Other Diseases in the Study Population

The dataset was composed of *n* = 77,115 cattle, with 21.8% of all heifers (9,167/42,009) and 16.3% of all steers (5,733/35,106) treated for any disease; consequently, 19.3% of cattle had any disease throughout the feeding cycle (14,900/77,115). Of the sick cattle, 6% were treated for DD (894/14,900), 59.1% for FR (8,804/14,900), 10.7% for BRD (1,590/14,900), 12.7% for OT (1,889/14,900), and 11.5% had TM diseases (1,723/14,900). Furthermore, in the TM category, 2.1% were also treated for DD (37/1,723), 49.3% for FR (849/1,723), 38% for BRD (655/1,723), and 10.5% (182/1,723) were treated for two other diseases, as seen in Table 1.

**Table 1.** Total number of cases (percentages) in each disease category, in data collected from two feedlots by type and sex (*n*=77,115 total animals and *n*=14,900 treated by any disease), for a 3-yr interval

	Type				Sex		Type × sex^1^							
	Calves		Yearlings				Calves				Yearlings			
Disease^2^	Fall-placed	Winter-placed	Grass fed	Background	Heifer	Steer	FPH	FPS	WPH	WPS	GYH	GYS	BYH	BYS
DD	120 (0.6)	318 (1.6)	158 (0.7)	298 (2.1)	642 (1.5)	252 (0.7)	37 (0.5)	83 (0.6)	249 (1.9)	69 (1)	79 (0.7)	79 (0.7)	277 (2.8)	21 (0.5)
FR	2,119 (10.2)	3,147 (15.5)	1,364 (6.2)	2,174 (15.6)	5,387 (12.8)	3,417 (9.7)	1,073 (13.8)	1,046 (8)	1,934 (14.5)	1,213 (17.6)	760 (6.8)	604 (5.5)	1,620 (16.7)	554 (13.1)
BRD	887 (4.3)	433 (2.1)	158 (0.7)	112 (0.8)	970 (2.3)	620 (1.8)	477 (6.1)	410 (3.1)	298 (2.2)	135 (2)	122 (1.1)	36 (0.3)	73 (0.7)	39 (0.9)
OT	616 (3)	602 (3)	364 (1.7)	307 (2.2)	1,121 (2.7)	768 (2.2)	262 (3.4)	354 (2.7)	429 (3.2)	173 (2.5)	247 (2.2)	117 (1.1)	183 (1.9)	124 (2.9)
TM	667 (3.2)	563 (2.8)	202 (0.9)	291 (2.1)	1,047 (2.5)	676 (1.9)	335 (4.3)	332 (2.5)	384 (2.9)	179 (2.6)	95 (0.8)	107 (1)	233 (2.4)	58 (1.4)
Total treated	4,409 (21.2)	5,063 (25)	2,246 (10.2)	3,182 (22.8)	9,167 (21.8)	5,733 (16.3)	2,184 (28)	2,225 (17.1)	3,294 (24.6)	1,769 (25.7)	1,303 (11.7)	943 (8.6)	2,386 (24.6)	796 (18.9)
Total animals	20,799 (100)	20,269 (100)	22,102 (100)	13,945 (100)	42,009 (100)	35,106 (100)	7,786 (100)	13,013 (100)	13,379 (100)	6,890 (100)	11,132 (100)	10,970 (100)	9,712 (100)	4,233 (100)

^1^FPH/FPS, fall-placed heifer/steer; WPH/WPS, winter-placed heifer/steer; GYH/GYS, grass fed yearling heifer/steer; BYH/BYS, background yearling heifer/steer.

^2^DD, digital dermatitis; FR, foot rot; BRD, bovine respiratory disease; OT, other; TM, two or more diseases.

FR was the disease with the highest cumulative incidence in both heifers and steers (58.8 and 59.6%, respectively). Of total FPC diagnosed with any disease, 48.1% of the cases were FR. From treated animals in the category WPC, 62.2% were treated for FR. Of all cattle treated in the GY category, 60.7% were treated for FR, and for BY, 68.3% of the total treated animals were diagnosed with FR.

Of cattle diagnosed as sick, BRD accounted for 10.6% and 10.8% of all cases in heifers and steers, respectively. For FPC, BRD accounted for 20% of all diseases diagnosed. In WPC, the proportion of treated cattle diagnosed with BRD was 8.5%. Category GY had 7% of all disease diagnoses as BRD, whereas for the BY category, BRD was responsible for 3.5% of all diseases diagnosed. Furthermore, BRD is predominantly prevalent in calves relative to yearlings, as seen in Table 2.

**Table 2.** Average cumulative incidence (mean ± SD) in DD, FR, and BRD, in data collected from two feedlots by type and sex (*n*=77,115 total animals and *n*=14,900 treated by any disease), for a 3-yr interval

Disease^1^		Cumulative incidence	SD^2^	CI^3^	
Calves					
	DD	137	30	102	171
	FR	1,698	600	1,019	2,377
	BRD	438	274	128	749
Yearlings					
	DD	156	38	113	198
	FR	1,430	395	983	1,877
	BRD	89	31	54	124

^1^DD, digital dermatitis; FR, foot rot; BRD, bovine respiratory disease.

^2^SD, standard deviation.

^3^CI, confidence interval.

Statistically significant differences were observed within types of cattle by sex, when comparing productive parameters of diseased cattle against healthy animals.

### Diseases and ADG

As shown in Table 3, some of the groups stratified by type and sex reported a statistically significant difference between ADG of healthy and DD-affected cattle. Healthy cattle had a significantly higher ADG compared with DD cattle in four of eight categories [fall-placed steer (**FPS**), winter-placed steer (**WPS**), winter-placed heifer (**WPH**), and grass fed yearling steer (**GYS**)], with the difference ranging from 0.11 to 0.17 kg/d. Further descriptive data on the ADG of healthy vs. DD cattle can be seen in Table 4.

**Table 3.** Average daily gain (kg/d) (ADG; mean ± SD) of cattle in each of the disease categories, by cattle type and sex

	Type × ^s^ex^1^							
	Calves				Yearlings			
Disease^2^	FPH	FPS	WPH	WPS	GYH	GYS	BYH	BYS
HE	1.35 ± 0.19^a^	1.57 ± 0.2^a^	1.5 ± 0.23^a^	1.73 ± 0.24^a^	1.56 ± 0.34^a^	1.79 ± 0.31^a^	1.49 ± 0.39^a^	1.69 ± 0.33^a^
DD	1.34 ± 0.18^*a,b,c*^	1.4 ± 0.17^*d*^	1.39 ± 0.22^*b,c*^	1.62 ± 0.23^*b*^	1.47 ± 0.31^*a,b,c*^	1.66 ± 0.33^*b,c*^	1.43 ± 0.24^*a,b*^	1.55 ± 0.24^*a,b*^
FR	1.35 ± 0.18^*a*^	1.58 ± 0.18^*a*^	1.49 ± 0.23^*a*^	1.75 ± 0.22^*a*^	1.51 ± 0.32^*b*^	1.77 ± 0.31^*a,b*^	1.43 ± 0.26^*b*^	1.68 ± 0.27^a^
BRD	1.3 ± 0.19^*b*^	1.53 ± 0.23^*b*^	1.42 ± 0.27^*b*^	1.64 ± 0.35^*b*^	1.42 ± 0.37^*b,c,d*^	1.64 ± 0.39^*a,b,c*^	1.33 ± 0.42^*b,c*^	1.53 ± 0.55^*a,b*^
OT	1.24 ± 0.21^*c*^	1.51 ± 0.21^*b*,*c*^	1.33 ± 0.34^*c*^	1.55 ± 0.27^*b*^	1.34 ± 0.4^*c,d*^	1.71 ± 0.36^*a,b,c*^	1.24 ± 0.3^*c*^	1.45 ± 0.45^*b*^
TM	1.24 ± 0.21^*c*^	1.46 ± 0.23^*c*,*d*^	1.35 ± 0.26^*c*^	1.59 ± 0.3^*b*^	1.27 ± 0.4^*d*^	1.62 ± 0.36^*c*^	1.29 ± 0.27^*c*^	1.43 ± 0.39^*b*^
Treated	1.31 ± 0.2	1.53 ± 0.2	1.44 ± 0.26	1.7 ± 0.26	1.45 ± 0.35	1.73 ± 0.33	1.39 ± 0.28	1.61 ± 0.34
All	1.34 ± 0.2	1.57 ± 0.21	1.49 ± 0.24	1.73 ± 0.25	1.55 ± 0.35	1.78 ± 0.31	1.47 ± 0.36	1.68 ± 0.33

^*a–d*^Within a column, values without a common superscript differed (*P* < 0.05).

^1^FPH/FPS, fall-placed heifer/steer; WPH/WPS, winter-placed heifer/steer; GYH/GYS, grass yearling heifer/steer; BYH/BYS, background yearling heifer/steer.

^2^DD, digital dermatitis; FR, foot rot; BRD, bovine respiratory disease; OT, other; TM, two or more diseases.

**Table 4.** Average daily gain (kg/d) (mean and 95% CI) of healthy vs. DD cattle, by cattle type and sex

	Healthy				DD^1^			
Type/sex^2^	Mean (kg/d)	SE^3^	95% CI^4^		Mean (kg/d)	SE	95% CI	
FPH	1.35	0.002	1.34	1.35	1.34	0.03	1.28	1.40
FPS	1.57	0.001	1.57	1.58	1.40	0.02	1.36	1.44
WPH	1.50	0.002	1.49	1.5	1.39	0.01	1.36	1.41
WPS	1.73	0.003	1.73	1.74	1.61	0.03	1.56	1.67
GYH	1.56	0.003	1.55	1.56	1.47	0.03	1.40	1.54
GYS	1.78	0.003	1.78	1.79	1.66	0.04	1.59	1.74
BYH	1.49	0.004	1.48	1.5	1.43	0.01	1.40	1.45
BYS	1.69	0.005	1.68	1.7	1.55	0.05	1.44	1.66

^1^DD, digital dermatitis.

^2^FPH/FPS, fall-placed heifer/steer; WPH/WPS, winter-placed heifer/steer; GYH/GYS, grass fed yearling heifer/steer; BYH/BYS, background yearling heifer/steer.

^3^SE, standard error.

^4^CI, confidence interval.

Healthy cattle had a significantly higher ADG compared with FR cattle in two of eight categories, ranging from 0.05 kg/d in grass yearling heifers to 0.06 kg/d in background yearling heifers (**BYHs**).

Healthy cattle had a significantly higher ADG compared with BRD cattle in six of eight categories [FPH, FPS, WPH, WPS, grass fed yearling heifer (**GYH**), BYH], with the difference ranging from 0.04 to 0.16 kg/d.

### Diseases and Partial Budget Analysis

Healthy cattle had a significantly higher partial budget compared with DD cattle in four of eight categories, ranging from $72 in winter-placed heifers to $98 in grass yearling heifers.

Healthy cattle had a significantly higher partial budget compared with FR cattle in two of eight categories, ranging from $3 in winter-placed heifers to $30 in BYHs.

Healthy cattle had a significantly higher partial budget compared with BRD cattle in six of eight categories, ranging from $74 in FPHs to $165 in background yearling steers. Descriptive data on the partial budget analysis can be seen in Tables 5 and 6.

**Table 5.** Average difference (healthy–disease) in the partial budget analysis ($CAD) by cattle type and sex

	FPH^1^	FPS	WPH	WPS	GYH	GYS	BYH	BYS
He–DD^2^	131^*^	–77.7	–72.5	3.5	–98.3	–95.8	10.7	–50.3
He–FR	–13.5	23.7	–3.3	14.9	4.9	–13.9	–30.4	–8.5
He–BRD	–73.9	–19.5	–99.2	–129.8	–148.4	–67.2	–142.8	–165.4

^1^FPH/FPS, fall-placed heifer/steer; WPH/WPS, winter-placed heifer/steer; GYH/GYS, grass fed yearling heifer/steer; BYH/BYS, background yearling heifer/steer.

^2^DD, digital dermatitis; FR, foot rot; BRD, bovine respiratory disease. ^*^Numbers in red are significantly different.

**Table 6.** Average partial budget ($CAD; mean and 95% CI) of healthy vs. DD cattle, by cattle type and sex

	Healthy				DD^1^			
Type/sex^2^	Mean ($CAD)	SE^3^	95% CI^4^		Mean ($CAD)	SE	95% CI	
FPH	1,509	3	1,502	1,514	1,639	41.3	1,556	1,723
FPS	1,656	2.3	1,651	1,660	1,578	25.5	1,527	1,629
WPH	1,660	2.3	1,656	1,665	1,588	12.6	1,563	1,613
WPS	1,837	3	1,831	1,843	1,840	21.6	1,797	1,883
GYH	1,800	2.5	1,796	1,805	1,702	25.4	1,652	1,753
GYS	1,878	2	1,874	1,881	1,782	25.5	1,731	1,833
BYH	1,750	2.4	1,745	1,755	1,761	11.7	1,738	1,784
BYS	1,938	3.4	1,931	1,944	1,887	51.7	1,779	1,995

^1^DD, digital dermatitis.

^2^FPH/FPS, fall-placed heifer/steer; WPH/WPS, winter-placed heifer/steer; GYH/GYS, grass fed yearling heifer/steer; BYH/BYS, background yearling heifer/steer.

^3^SE, standard error.

^4^CI, confidence interval.

Furthermore, information on the DOF for the diverse categories is summarized in Tables 7 and 8.

**Table 7.** Days on feed (mean ± SD) of cattle in each of the disease categories, by cattle type and sex

Type × sex^1^								
	Calves				Yearlings			
Disease^2^	FPH	FPS	WPH	WPS	GYH	GYS	BYH	BYS
HE	271 ± 50^*b*^	265 ± 47^*d*^	212 ± 50^*b*^	207 ± 41^*b,c*^	162 ± 29^*b,c*^	164 ± 23^*b*^	173 ± 19^*b*^	155 ± 43
DD	298 ± 47^*c*^	292 ± 57^*e*^	232 ± 36^*c*^	210 ± 30^*b,c*^	170 ± 16^*c*^	160 ± 21^*a,b*^	182 ± 12^*c*^	150 ± 30
FR	270 ± 41^*b*^	252 ± 46^*b,c*^	214 ± 44^*b*^	192 ± 33^*a*^	162 ± 19^*b,c*^	157 ± 22^*a*^	179 ± 19^*c*^	160 ± 37
BRD	268 ± 38^*a*^	246 ± 35^*a,b*^	235 ± 43^*c*^	197 ± 38^*a,b*^	153 ± 29^*a*^	166 ± 20^*a,b*^	169 ± 31^*b*^	152 ± 45
OT	258 ± 44^*a*^	242 ± 44^*a*^	196 ± 54^*a*^	199 ± 54^*a,b*^	150 ± 26^*a*^	155 ± 23^*a*^	158 ± 29^*a*^	156 ± 48
TM	262 ± 42^*a,b*^	261 ± 46^*c,d*^	229 ± 42^*c*^	202 ± 41^*a,b*^	155 ± 25^*a,b*^	156 ± 24^*a*^	177 ± 19^*b,c*^	161 ± 34
Treated	267 ± 42	252 ± 45	217 ± 46	195 ± 35	159 ± 23	157 ± 22	177 ± 21	159 ± 39
All	270 ± 48	263 ± 47	213 ± 49	204 ± 40	162 ± 29	163 ± 23	174 ± 20	156 ± 42

^a–d^Within a column, values without a common superscript differed (*P* < 0.05).

^1^FPH/FPS, fall-placed heifer/steer; WPH/WPS, winter-placed heifer/steer; GYH/GYS, grass fed yearling heifer/steer; BYH/BYS, background yearling heifer/steer.

^2^DD, digital dermatitis; FR, foot rot; BRD, bovine respiratory disease; OT, other; TM, two or more diseases.

**Table 8.** Days on feed (DOF; mean and 95% CI) of healthy vs. DD cattle, by cattle type and sex

	Healthy				DD^1^			
Type/sex^2^	Mean (d)	SE^3^	95% CI^4^		Mean	SE	95% CI	
FPH	271	1	269	272	298	8	283	314
FPS	265	1	264	266	292	6	280	305
WPH	212	1	211	213	232	2	228	237
WPS	207	1	206	208	210	4	203	217
GYH	162	1	162	163	170	2	167	174
GYS	164	1	163	164	160	2	155	165
BYH	173	1	173	174	182	1	181	184
BYS	155	1	154	157	150	7	136	164

^1^DD, digital dermatitis.

^2^FPH/FPS, fall-placed heifer/steer; WPH/WPS, winter-placed heifer/steer; GYH/GYS, grass fed yearling heifer/steer; BYH/BYS, background yearling heifer/steer.

^3^SE, standard error.

^4^CI, confidence interval.

## DISCUSSION

Our findings placed FR as the disease with the greatest treatment cumulative incidence (59%), followed by BRD (10.7%) and DD (6%). However, it is well known that BRD is the most prevalent health problem in North American feedlot cattle, ranging from 30% to 75% of total feedlot morbidity (Griffin, 1997; Brooks et al., 2011), with lameness being the second most prevalent condition (Schwartzkopf-Genswein et al., 2016). Recently, a dataset analysis of 28 feedlots over a decade of data showed a BRD treatment cumulative incidence of 50.4%, whereas for FR it was 20.7% (Davis-Unger et al., 2019). The differences in the cumulative incidences reported could be attributed to multiple factors at cattle (weaning process, lack of immunity, type of animals entering the feedlot) and management practice (pooling of cattle from different sources, transportation, weather, recording optimization) (Taylor et al., 2010). However, a more detailed description on risk factors is hindered by the lack of data on calf processing, given that comparing solely weight at arrival would provide only a partial explanation. Another explanation might be that pen-level treatment for diseases that are not taken into account in our data only included individual animal-level treatments.

In the present study, a single case of DD negatively affected ADG and the partial budget, with the magnitude dependent on cattle type and sex. Few studies have examined production and economic impacts of a single case of DD relative to HE in feedlot cattle.

### Economic Impacts of Infectious Diseases

DD affected the partial budget in five out of the eight groups of cattle, with the highest impact of DD seen in GYH and GYS ($-98 and $-96 CAD, respectively, relative to their healthier counterparts). Also, in the group FHC, those with DD had a significantly higher partial budget than HE, attributed to DD cattle having a longer DOF (+27) and a relatively higher TW, with negligible impact on the feed cost.

In addition, we found statistically significant difference in the partial budget between HE and FR in feedlot cattle in only two categories, where previous research reported no difference (Davis-Unger et al., 2018). This can be explained by the relative ease of FR field diagnosis, symmetrical swelling of the foot, and sudden and severe lameness, with rapid resolution expected if treatment is initiated soon after onset (Metre et al., 2005), thereby limiting its negative impact.

For BRD, in six of the eight groups, HE had a higher partial budget, placing BRD as the costliest of the three diseases, with DD being in second place and finally FR in third. This was partially supported by the literature, as BRD had the most impact of diseases that emerged in a feedlot (Jim, 2009; Cernicchiaro et al., 2013; Leach et al., 2013). In contrast, for DD, economic impact of a single case in feedlot cattle had not been reported. In dairy, it was estimated that a single case of DD or FR costs $US 128 (van Amstel and Shearer, 2006), attributed to additional value of milk and reproduction, relative to the beef system.

Nevertheless, it is important to acknowledge that the current ranking is based on an individual level comparison of diseased vs. healthy animals and not necessarily at a population level. That is to say, BRD is the costliest of the three diseases relative to a healthy animal; however, in yearlings, in which the cumulative incidence of BRD is lower than that in calves, this may no longer be the costliest disease at a population level.

 In addition, purchase price might have influenced cattle performance, as it reflects cattle quality, therefore being a limitation of the current study.

### ADG

Healthy cattle had a significantly higher ADG compared with DD cattle in five of the eight categories (ranged from 0.11 kg/d in WPC heifers to 0.17 kg/d in FPC steers). It has been reported that sick cattle experience a decrease in feed intake and number of visits to the feed bunk, due to their decreased ability to compete with nonsick cows (Jensen and Proudfoot, 2017). However, the impact of diseases on ADG varied by cattle type and sex, as reported in the current study.

It has also been reported that steers with active lesions (M2) gained 0.08–0.14 kg/d less than healthy cattle with no active lesions (Kulow et al., 2017). However, in our study, we did not score M-stages; therefore, no comparison was performed at that level.

For cattle diagnosed with FR, it was documented by Tibbetts et al. (2006) that a single FR incidence caused 0.03 kg/d less, relative to HE (1.27 vs. 1.3 kg/d, respectively). The fact that several categories of animals with FR did not report a decrease in the productive parameters can be explained by a quick diagnosis, treatment, and healing, and in doing so it decreases its negative impacts.

Cattle diagnosed with BRD had a significantly lesser ADG relative to HE in six of the eight categories, ranging from 0.04 kg/d in FPC steers to 0.16 kg/d in BY heifers. Those findings were in agreement with previous studies, in which a reduction in the ADG of cattle affected by BRD was estimated to be between 0.07 kg/d (Schneider et al., 2009) and 0.09 kg/d (Griffin, 2014) less than healthy animals without BRD. Therefore, BRD is still an important economic driver of lost revenue and warrants further investigation for its control and mitigation.

A variation of the effect of the disease on the ADG was expected across different types of cattle, as it is influenced by sex and age, as evidenced with DD, FR, and BRD. However, it is still unclear what is causing this decrease in the ADG, whether it is an increase in the energy expenditure for movement (Dijkman, 1997), access to the feed-bunk, and/or decrease of the feed intake, and how this is affected by pen condition.

### Limitations

Retrospective database analysis always has limitations and potential biases; this study is no exception. Despite a large number of animals included, the number of feedlots included limits the external validity. However, the inclusion of diseases like FR or BRD allows for a better understanding of the relative impact of DD in feedlot cattle as more data are available in literature on impact of these two disorders.

Although the feedlot crew was trained on case identification by the vet clinic, there is always the risk of misdiagnosis. For example, DD disease diagnosis is not easily done in the field, mainly due to a lack of clinical signs, where not all DD results in lameness, and due to the area of the foot affected not always available for visual inspection. Furthermore, the chronic nature of this disease (Krull et al., 2016a) may allow it to go unnoticed for long intervals, causing an underestimation of its cumulative incidence and perhaps greater effects on production parameters, relative to acute diseases such as FR, as noted in this study. This might increase the risk of differential misclassification, causing an underestimation of the presence of DD and, eventually, its economic impact. With the disease emerging, routine checkups are advised, where the ideal method is a close-feet inspection that might imply lifting the legs.

Furthermore, although the current evaluation only included single case events for DD, FR, and BRD purposively, recrudescence of those infectious diseases has been widely described in domestic animals (O’Connor et al., 2013; Palmer and O’Connell, 2015; Clifton and Green, 2016); therefore, there is the possibility of an underestimation of the production and economic impact. It is also worth noting how cases of DD, FR, and BRD were present in the TM category, which contributes to a miscalculation and underestimation of the real impact of each disease, nonetheless, representing how impactful they can be in a herd.

Also, the higher prevalence of FR relative to BRD differs from what is commonly reported, where BRD is placed as the most common disorder. However, a more detailed explanation of this phenomenon is hindered by the lack of animal and environmental data in the early production and transitional stages of feedlot cattle.

In addition, dressing percentage has been documented to be influenced by genetic and non-genetic factors (diet, age, gut fill, sex, breed and within breed differences); therefore, the estimation of the slaughter weight could be oversimplified by the lack of detailed data at the individual level.

The cost of the calf was not included, given that these data were not available for cattle in the database. This limits the evaluation of the influence of cattle quality (as reflected by purchase price) in the risk of getting diseases.

As the ranking was done at an animal-level comparison of disease vs. healthy, population-level evaluations may yield different results depending on the cumulative incidence.

## CONCLUSION

DD is causing a decrease in the ADG of some categories of feedlot cattle, where no significant differences were observed between DD and the impact of BRD in the ADG of cattle in seven out of the eight categories.

This is the first report to investigate not only production but also economic impacts of a single case of DD in Canadian feedlot cattle. The differential economic impacts based on type of cattle and sex allow for the creation of strategies focussed on specific groups that are more predisposed resulting in largest economic impact. Further studies are needed to evaluate various strategies to deal with infectious diseases that cause lameness, by developing a cost-effectiveness analysis. This would provide producers with more management tools suited to a feedlot setting.

Identifying the most impactful conditions in feedlot cattle production systems could optimize mitigation and management strategies, prompting focus on specific subpopulations more susceptible to disease occurrence and ultimately to understand dynamics of budget analysis. The creation and implementation of an early detection system for DD, suitable for a feedlot setting, are of paramount importance. This combined with group-level interventions, e.g., improvement in pen condition and the use of footbaths, could help to mitigate and control this disease, allowing better outcomes from the economic, productive, and welfare perspectives.
